# Boosting grapevine breeding for climate-smart viticulture: from genetic resources to predictive genomics

**DOI:** 10.3389/fpls.2023.1293186

**Published:** 2023-12-11

**Authors:** Gabriele Magon, Valeria De Rosa, Matteo Martina, Rachele Falchi, Alberto Acquadro, Gianni Barcaccia, Ezio Portis, Alessandro Vannozzi, Emanuele De Paoli

**Affiliations:** ^1^ Department of Agronomy, Food, Natural Resources, Animals and Environment (DAFNAE), Laboratory of Plant Genetics and Breeding, University of Padova, Agripolis, Viale dell’Università 16, Legnaro, Italy; ^2^ Department of Agricultural, Food, Environmental and Animal Sciences (DI4A), University of Udine, Via delle Scienze, 206, Udine, Italy; ^3^ Department of Agricultural, Forest and Food Sciences (DISAFA), Plant Genetics, University of Torino, Largo P. Braccini 2, Grugliasco, Italy

**Keywords:** biotic and abiotic resistance, grapevine, genetic resources, genomic resources, vitis, resilient traits, genomic and phenomic prediction

## Abstract

The multifaceted nature of climate change is increasing the urgency to select resilient grapevine varieties, or generate new, fitter cultivars, to withstand a multitude of new challenging conditions. The attainment of this goal is hindered by the limiting pace of traditional breeding approaches, which require decades to result in new selections. On the other hand, marker-assisted breeding has proved useful when it comes to traits governed by one or few genes with great effects on the phenotype, but its efficacy is still restricted for complex traits controlled by many loci. On these premises, innovative strategies are emerging which could help guide selection, taking advantage of the genetic diversity within the *Vitis* genus in its entirety. Multiple germplasm collections are also available as a source of genetic material for the introgression of alleles of interest via adapted and pioneering transformation protocols, which present themselves as promising tools for future applications on a notably recalcitrant species such as grapevine. Genome editing intersects both these strategies, not only by being an alternative to obtain focused changes in a relatively rapid way, but also by supporting a fine-tuning of new genotypes developed with other methods. A review on the state of the art concerning the available genetic resources and the possibilities of use of innovative techniques in aid of selection is presented here to support the production of climate-smart grapevine genotypes.

## 1 Introduction

Grapevine (*Vitis vinifera* L.) is a highly cultivated crop worldwide, encompassing an extensive surface area of 7.3 million hectares and yielding approximately 71 million tons of berries annually. With a domination of 47.4% in the viticulture sector, wine is the primary derivative product, followed by table grapes (44.5%) and raisins (8%) (OIV, 2022). This kind of production is under direct threat from the impact of climate change on the agricultural productive systems. The evident impacts of anthropogenic activities increased the frequency and severity of droughts, and the occurrence of extreme catastrophic events, finally resulting in a profound influence on natural ecosystems and on the agricultural sector as a whole. These effects have raised concerns about sustaining and improving crop productivity (USGCRP, 2018). Clearly, the viticulture and wine industry are not exempt to these issues: as extensively demonstrated, climate and weather have a substantial impact on viticultural productivity, both from a quantitative and qualitative point of view (Jones et al., 2022). This peculiar impact that the environmental factors exert on the production is encompassed in the concept of “terroir”, which represents the specific signature that the combination of climate, soil and agronomical practices confers to the quality of grapes, and ultimately wine (Perin et al., 2020; Fernández-Marín et al., 2013). In this context, climate changes are a real threat for all the specific combinations of aromatic and organoleptic compounds constituting the unicity and the typicity of the designation of origin productions. Since three to four decades ago climate change has had a substantial impact on grapevine output, and although some viticultural techniques or cultivation zones may be able to manage this impact at least temporarily, long-term plans must be implemented for some other areas (Delrot et al., 2020). To this purpose, conventional breeding techniques (CBTs) and new breeding techniques (NBTs) represent two powerful tools to cope with climate change in an optic of low input productive systems. CBTs and NBTs are substantially addressed to the genetic improvement of both rootstock and scion. Based on the objectives being pursued, breeding efforts vary, as summarized by Delrot et al. (2020). However, in the context of climate change, all of these objectives primarily revolve around enhancing nutrient utilization efficiency and developing resistance or tolerance to abiotic stresses, pests, and pathogens. In contrast to historical and cultural legacies that see viticulture as a conservative discipline, in recent years the importance of conventional genetic improvement is gradually rising up (Vannozzi et al., 2021).

## 2 Genetic resources and agrobiodiversity

As previously mentioned, the global vineyard surface spans 7.3 million hectares, with the 13 most cultivated varieties accounting for 2.8 million hectares (37.3%) (OIV, 2017; OIV, 2022). Considering the estimated global count of approximately 10,000 grapevine varieties (Galet, 2000), the aforementioned data is even more relevant. A pronounced imbalance exists between the cultivars and clones cultivated worldwide, and the actual available biodiversity. Maintaining and preserving high levels as it provides a reservoir of genetic heterogeneity is crucial as it provides a reservoir of valuable allelic combinations that can offer genetic resistance or tolerance to both biotic and abiotic stresses. These resources are highly advantageous for breeding programs and their development. It is indeed crucial to implement measures focused on safeguarding grapevine biodiversity. In this regard, conservation can be achieved through two main strategies: the *in-situ* conservation and the *ex-situ* conservation. Regarding the first conservation strategy, the landrace-based orchards in inherent regions represent biodiversity hotspots that preserve natural resources like soil fertility, air and landscape quality because of the ideal genotype-environment interactions that permit ecologically benign agronomic techniques. The establishment of formal institutions and protected areas ensure the safeguarding of local biodiversity by preventing habitat destruction caused by various events such as embankment management, street edge cleaning, forest cutting and fires. In this regard, the study by Biasi and Brunori (2015) extensively explores the agroecosystem of Grechetto Rosso, a grapevine landrace from the Bolsena lake hills in Lazio (Central Italy). In particular, both the landrace-based vineyard patch structure and the surrounding vineyard landscape were examined for shape, complexity, and heterogeneity of the margins using data on landscape pattern, configuration, and composition at large and detailed scales. It is necessary to specify that this kind of activity is underestimated in its magnitude. In this regard, a lot of dedicated caretaker farmers and several stakeholders contribute diligently to the safeguard of biodiversity through the application of sustainable agriculture practices, aimed to the protection of a wide network system of conservation vineyards. Unfortunately, the vast majority of these remains invaluable and unknown, provoking an underappreciation of their incidence within agricultural, environmental and scientific communities. On the other hand, *ex-situ* conservation aims to constitute a collection of germplasm characterized by high genetic diversity, but in a different area from the original. This strategy has the advantage of conducting more rigorous management and direct supervision of biological material. It allows comparative studies on phenological models and evaluation of resistance/tolerance to biotic and abiotic stresses within a single environment. A clear example of that is the *Vitis* germplasm repository of CRA-VIT of Conegliano (Consiglio per la Ricerca e la Sperimentazione in Agricoltura, Centro di Ricerca per la Viticoltura), in Veneto (North Eastern Italy). This collection comprises over 3600 accessions, encompassing twenty different species belonging to the *Vitis* genus. The reported number of accessions should be viewed as very dynamic, with new accessions being annually added and existing ones being eliminated due to improved redundancy detection. These plants have been characterized and evaluated based on their morphological, physiological, biochemical, genetic and agronomic characteristics (Gardiman and Bavaresco, 2014). It is worth noting that the safeguard of germplasm has long been a central issue. Yet in 1998, at a time when microsatellites profiling had not yet widespread, the structure of a grapevine collection composed by 67 accessions sited in Logroño (La Rioja, Northern Spain) was analyzed by AFLP marker profiles. The analysis provided the first insights into DNA profiling for the efficient management of germplasm collections (Cervera et al., 1998). In general, the molecular analysis of population genetic structure is a consistent aspect across plant germplasm collection initiatives. It allows indeed to deepen the various stratification levels explaining the diversity among cultivars, but also between domesticated grapevines and wild relatives. In this regard, Emanuelli et al. (2013), using a molecular markers panel composed of 22 SSRs (Single Sequence Repeats) and 384 SNPs (Single Nucleotide Polymorphism), deciphered the structure of 1659 cultivated grapevines (*V. vinifera* ssp. *sativa*), 177 wild individuals of *V. vinifera* ssp. *sylvestris*, 127 interspecific hybrids used for fruit production and 310 accessions of rootstock varieties including wild non-vinifera *Vitis* species. The study was conducted in San Michele all’Adige (Trentino-Alto Adige, Northeastern Italy). The results allowed the identification of homogeneous clusters based on the genetic similarity, reflecting the evolutionary history of a cultivar/species with respect to another. Moreover, these findings were precious to avoid redundancy in germplasm collection guaranteeing its practical utility. Scientific literature is full of studies conducted in this regard. As an example, Lacombe et al. (2013) focused its effort on the grapevine germplasm repository of Domaine de Vassal (INRAE, France). A total number of 2344 unique *V. vinifera* accessions were analyzed with a panel of 20 SSRs and a parentage study was carried out, clarifying the breeding history and the genetic constitution of cultivated grapevine. Results highlighted main genitors involved in varietal assortment evolution. On the same wavelength, something similar was performed by Klein et al. (2018), which used a set of 11,200 SNPs to reconstruct the phylogenetic relationships between more than 300 accessions of 24 *Vitis* species (12 North American species, 7 Eurasian species) and 4 *Ampelopsis* species from living germplasm collections maintained by the USDA-ARS Plant Genetic Resources Unit and the National Clonal Germplasm Repository (Davis, California, USA). In this case, in addition to shed light on the phylogenetic relationships occurring among the different *Vitis* species, it was also possible to correctly catalog 28 misidentified accessions and to systematically classify another 20 previously unknown. From this, it can be seen that this type of analysis not only enables direct varietal recognition, but also allows clarification in situations of confusion generated by homonymy and synonymy. Frequent are indeed the cases in which it was discovered that what were previously considered as distinct varieties are, in fact, the same. In a study conducted on a gene bank composed of 621 accessions, an integrated characterization was given using morphological descriptors, isoenzymes and microsatellites. At the end of the analysis, it was discovered that the real number of unique accessions was 177, almost 30% of the initial number (Ortiz et al., 2004). The recognition of synonyms and homonyms is quite frequent in germplasm collection studies since cultivars nomenclature often originates from ancient popular culture and may vary across regions due to local languages and dialects. Clarifying these naming issues is important since it helps to preserve these genotypes and their contribution to grapevine diversity. Regarding this matter, Gago et al. (2022) conducted an ampelographic characterization of seven grapevine local varieties from Valencian Community (Spain), resolving certain misunderstandings that arose between the names of these varieties in Valencian language and the evolution that those names had over time. An analogous situation has been found in a collection of 61 *Vitis* accessions of Bucharest Faculty of Horticulture (Romany). Thanks to SSRs, it was possible to assess that some cultivars historically considered distinct and named in a completely different way, actually are the same grapevine variety (Popescu et al., 2017). The matter of naming is particularly relevant with regard to minor and local varieties. In a study conducted on 178 grapevine accessions, 62 correspond to typical varieties of Emilia-Romagna (Northern Italy). Among these 62, the SSR profile of 42 did not match with any reference and their name is known only for ancient documents or for oral transmission in Emilian or Romagna languages (Pastore et al., 2020). Most of them are at risk of extinction and the conservation in regional repositories, together with a molecular and phenotypic description which unequivocally identify each cultivar, helps to protect, maintain and propagate a germplasm of high genetic value. The management and the study of germplasm collections does not regard only actions operating at local or national level as reported so far (**Table 1**), but, embracing a collaborative spirit, has the potential to generate supranational initiatives. This is the case of Europe, where each country maintains its own varieties catalog with different descriptor parameters. Recognizing the need to address the issue of heterogeneity, and establishing standardized evaluation criteria, the GrapeGen06 project was launched. This collaborative research consortium aimed to create the European Vitis Database (www.eu-vitis.de), an integrated and interactive platform where curators can upload and modify their passport, characterization, SSR-markers, pathology-related data and photos for different grapevine varieties. The main aim is to safeguard and enhance the germplasm by monitoring its preservation via the creation of an accurate list of the European grapevine resources, firstly by merging the national catalogs (Lacombe et al., 2011), and then by refining through the correction of homonymy and misnaming cases, gaining in this way a univocal correspondence between a cultivar name and a genotype. This can be pursued by applying some standardized evaluation criteria, which includes the combination of phenotypic and phenological descriptors along with molecular markers, particularly conventional SSRs loci (Maul et al., 2012). As a result, the project successfully cataloged more than 32,400 accessions, which are maintained at different levels in 35 germplasm collections across 22 European countries. The novelty of this database is the possibility to update and interactively modify by the collection holders, thus making the whole system more flexible. This comprehensive and collaborative approach ensures a unified and accessible database for the study and preservation of germplasm resources in Europe. These new and common guidelines for *Vitis* germplasm characterization gave a boost even outside of Europe, also from a socioeconomic point of view. In Israel, for example, the standards established by the European Vitis Database were utilized to retrieve, census and characterize grapevine germplasm, in order to re-establish indigenous and traditional local varieties within the modern international wine industry, suffering a prolonged period of decline due to socio-religious reasons (Drori et al., 2017). The philanthropic aspects of initiatives like these are very interesting, in terms of help, support and solidarity to countries with developing economies. In this regard, special mention deserves the international research consortium, financed by the Government of Luxembourg, which led to the identification, collection, characterization and conservation of grapevine genetic resources across several Caucasus and Black Sea area countries, with the aim to improve local viticulture and winemaking industry (Maghradze et al., 2006). Germplasm collections are valuable also for intra-varietal comparison studies, such as trials in field conditions to test the susceptibility of different cultivars to the main diseases, e.g. downy and powdery mildew (Pavloušek, 2012 and Pavloušek, 2007), but also in case of abiotic stress evaluation, for example drought stress resistance in different rootstock hybrids (Pavloušek, 2011). In these cases, the availability of a germplasm collection of can be very useful, allowing the evaluation of the behavior of different varieties, under the same field conditions, at the same time.

**Table 1:** Summary of main germplasm collections reviewed.

Population	Number of accessions	Descriptors	Geographic area	Reference
*Vitis* spp.	3600	SSR	Veneto, Italy	Gardiman and Bavaresco, 2014
*Vitis vinifera*	67	AFLP	La Rioja, Spain	Cervera et al., 1998
*Vitis* spp.	2273	SSR and SNP	Trentino - Alto Adige, Italy	Emanuelli et al., 2013
*Vitis vinifera*	2344	SSR	Occitania, France	Lacombe et al., 2013
*Vitis* spp. and *Ampelopsis* spp.	300	SNP	California, USA	Klein et al., 2018
*Vitis vinifera*	621	SSR	Comunidad de Madrid, Spain	Ortiz et al., 2004
*Vitis vinifera*	7	Ampelographic	Comunitat Valenciana, Spain	Gago et al., 2022
*Vitis* spp.	61	SSR	Muntenia, Romany	Popescu et al., 2017
*Vitis vinifera*	178	SSR	Emilia - Romagna, Italy	Pastore et al., 2020

## 3 Genomic resources: sequencing and resequencing

**Figure 1:**
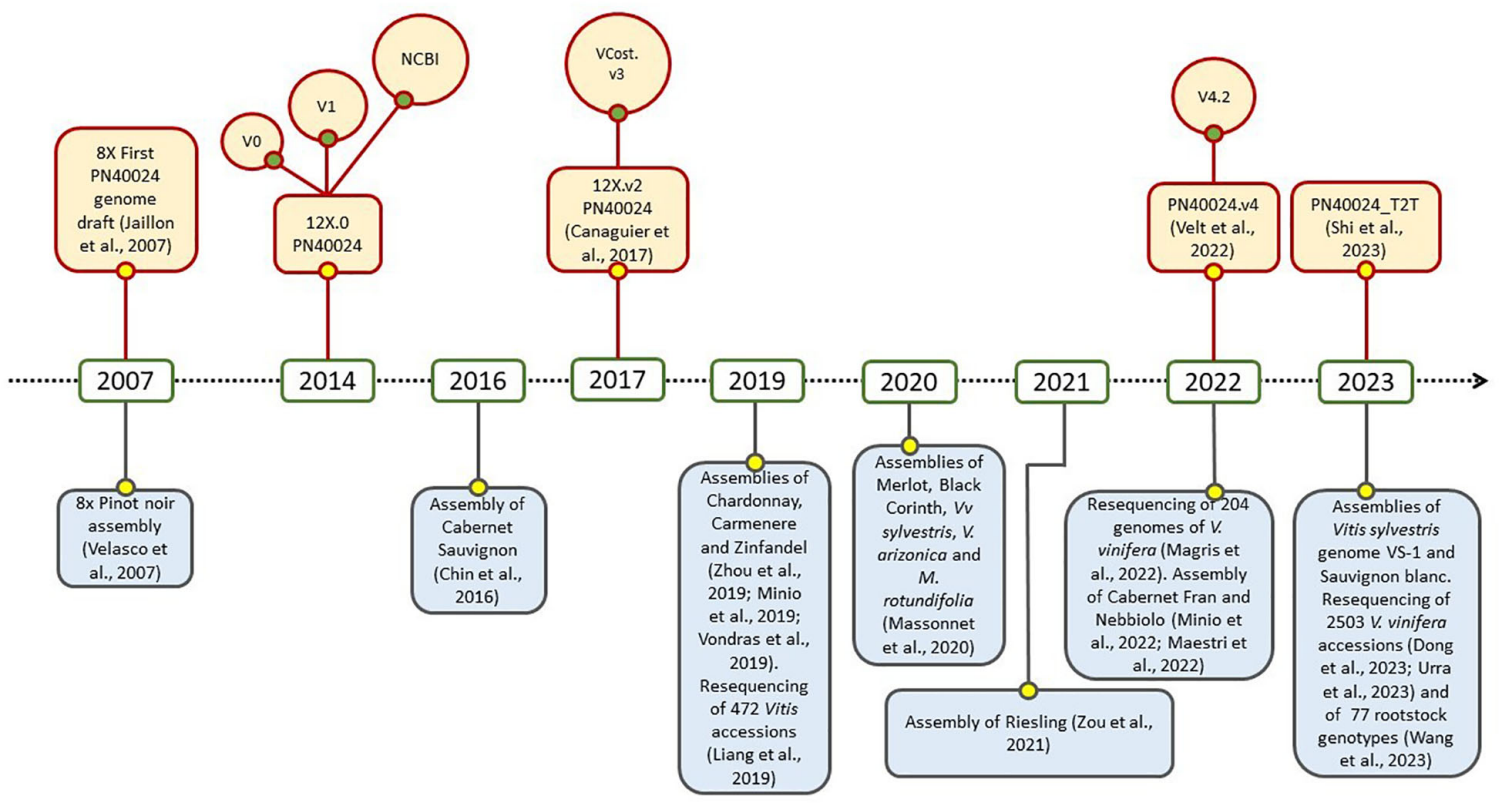
Timeline of the main sequencing and resequencing initiatives in grapevine: PN40024 (upper part) and other cultivars and *Vitis* spp. (lower part) history.

Due to its economic, cultural, and scientific importance, the draft genome sequencing for grapevine marked a series of milestones in the field of genomics, it was the first for a fruit crop, the second for a woody species, and the fourth for flowering plants. Two different drafts were published in 2007 by Velasco et al. and by the French–Italian Public Consortium for Grapevine Genome Characterization (Jaillon et al., 2007). While the first was obtained from a highly heterozygous accession of Pinot noir, the second one was assembled on the experimental inbred line called “PN40024”, in first instance believed deriving from Pinot noir cultivar, and selfed till a very high percentage of homozygosity (around 93%). At the beginning, the sequencing and the assembly were featured by an 8X coverage, but subsequently they were enhanced to 12X with an improvement in gene prediction. This last was precisely released in two versions: the 12X.v0, achieved at Genoscope in Evry (France; http://urgi.versailles.inra.fr/Species/Vitis/Data-Sequences/Genome-sequences; FN597015-FN597047 at EMBL, release 102), and the 12X.v1, performed at CRIBI in Padova (Italy) by merging the v0 with a gene prediction conducted with JIGSAW software (Allen and Salzberg, 2005; Forcato, 2010). An important contribution was given by Grimplet et al. (2012), with a comparative analysis which resulted in an efficient functional annotation of the predicted genes in the new assembly, and by Vitulo et al. (2014) for the discovery of splicing variants. In 2017, the third version of PN40024 assembly, namely 12X.v2, was released taking advantage of six dense parental genetic maps and a large anchoring effort (Canaguier et al., 2017). In this case, the annotation (called VCost.v3) was the result of the integration of the three previous annotations, namely NCBI Refseq, CRIBIv1 and Vcost. This effort was possible thanks to the International Grapevine Genome Program (IGGP) which operated within the COST Action FA1106. Recently, Velt et al. (2023) took a further step with the achievement of the fifth genome assembly, precisely PN40024.v4, and its related gene annotation PN40024.v4.2. The assembly was sensibly improved by combining the top-quality Sanger contigs of the 12X and the long reads sequencing (Single-Molecule Real-Time SMRT sequencing, PacBio). Interestingly, it was finally clarified that the original PN40024 did not originate from Pinot noir, but from Helfensteiner cultivar after several cycles of selfing. A further significant achievement in this field was made by Shi et al. (2023), with the publication of the telomere-to-telomere PN40024 genome (PN_T2T). By using PacBio HiFi long reads, it was possible to assemble a gap-free reference genome, 69 Mb longer and with 9018 additional genes. Moreover, 67% of the repetitive sequences, among 19 centromeres and 36 telomeres, were annotated. Overall, PN40024 represents a milestone for the entire scientific community working on grapevine and it is still an essential resource on which studies on genetics, genomics and transcriptomics of grapevine are based (**Figure 1**). On the other hand, the advent of third generation sequencing, which enabled the assembly of diploid genomes, and the necessity to directly study non-reference cultivars, led to the widespread of several genomes of wine grape varieties and also wild relatives (**Figure 1**), like Cabernet Sauvignon, Cabernet Franc, Zinfandel, Chardonnay, Nebbiolo, *Vitis riparia* and many others (Zhou et al., 2019; Massonnet et al., 2020; Maestri et al., 2022; Minio et al., 2022). In this regard, an important contribute was given by the study of Magris et al. (2021), in which the massive release of 204 sequenced genomes of *V. vinifera* highlighted that its evolutive history was featured by a single domestication event that occurred in Western Asia, and by subsequent, numerous and pervasive introgressions from European wild populations. Conversely, the recent analysis of 3.525 accessions led to the conclusions that two domestication events might have taken place concurrently in Western Asia and Caucasus, originating table and wine grapevines (Dong et al., 2023). It is noteworthy to point out that all these released genomes and annotation, together with many other genomic resources, are available and publicly consultable at GRAPEDIA portal (https://grapedia.org/) which currently is the last and more updated platform for grapevine. The GRAPEDIA initiative has the main goal to provide the grapevine scientific community with a single open-access portal, allowing data exploration and visualization of all resources, with tools for comparative analysis and customized services.

## 4 Grapevine cross breeding populations

Historically, crossbreeding in grapevine assumed a pivotal importance for resistance transfer from the wild relatives to the winemaking cultivars, in order to preserve the high quality of European varieties while equipping them with genetic resistances. This process, named introgression, involves crossing a wild individual with the resistance trait (the donor plant) with one from a winemaking variety (the receiver plant). After that, the recipient plant undergoes several backcrosses along with recurrent selection of the desired character. For a woody crop like grapevine with a lengthy juvenile period, this approach is labour and time-intensive without the certainty of a desirable outcome. From the first cross until the release of a new grapevine variety, on average 25-30 years pass (Eibach and Töpfer, 2015). Due to the vegetative propagation which rules in viticulture, every single seedling generated by cross breeding could potentially represent a new variety. For this reason, the initial focus is evaluating fundamental traits, which are largely determined by the intended use of the new cultivar. However, the most common trait assessed is typically the resistance to major pathogens. Then, in correspondence of the first flowering, the attention is focused on morphological traits, such as cluster architecture, shoot growth and axillary formation (Eibach and Töpfer, 2015). After this sorting, the selected plants are vegetatively propagated and the evaluation of quality determinants (aromatic compounds, secondary metabolites etc.) is carried out. Finally, during the last step before the new variety release, the most promising breeding lines undergo several trials to test the agronomic performance in different locations and different environments, by also investigating the interactive relationships between genotype and environment (Eibach and Töpfer, 2015). As it is immediately understandable, the procedure is long and complex and, in this regard, marker assisted breeding (MAB) could represent a valid strategy to speed up the process and to operate a more targeted and efficient selection.

### 4.1 Genotyping tools and genetic maps

The advance of molecular biology moved the breeding process from a pure empirical work to a targeted management of cross and a strictly goal oriented gene organization (Töpfer et al., 2011). In this regard, the arrangement of genes on corresponding chromosomes, which is established through recombination events between different genetic markers, is known as genetic map. The progressive development of a multitude of molecular markers allowed the refinement of genetic maps (Pirrello et al., 2023). SSRs, Random Amplified polymorphic DNA (RAPD), Amplified Fragment Length Polymorphism (AFLP), RFLP (Restriction fragment length polymorphism), Sequence Characterized Amplified Regions (SCARs) and SNP were over time exploited to accomplish dense genetic maps, aimed at the detection of QTLs (Quantitative Trait Locus) for specific traits of interest (Adam-Blondon et al., 2004; Fischer et al., 2004; Welter et al., 2007; Emanuelli et al., 2013). In order to determine the relative distribution of molecular markers across the chromosomes, genetic mapping takes advantage of the Mendelian laws of allelic assortment and recombination. Linkage maps are thus produced by evaluating the distance between two nearby loci by using genetic recombination frequencies. The first grapevine genetic map was published in 1995 by Lodhi et al. and it was built up from a single interspecific hybrid population of Cayuga White × Aurore. It is based on 422 RAPD and 16 RFLP markers which enabled, in this sense for the first time, QTLs localization and map-based gene cloning. From then on, a multitude of studies and research have been conducted, till the publication of more than 160 different grapevine genetic maps, each of them based on one type or more of molecular markers. In this regard, SSRs are still the best markers to compile genetic maps and understand genotype-phenotype relationships due to their stability and codominance, and moreover for the high transferability among grapevine genotypes (Vezzulli et al., 2019; Pirrello et al., 2023). The first two dense SSRs based genetic maps are those developed by Riaz et al. (2004) and Adam-Blondon et al. (2004). In the first case, a mapping population of full-sib, deriving from *V. vinifera* cvs. Riesling and Cabernet Sauvignon was used to construct a map of 181 informative microsatellite markers. In the second case, the number was raised to 245 by constructing the reference map based on a mapping population derived from crosses between *V. vinifera* cvs. Syrah and Grenache, and another population derived from the reciprocal cross. Further improvements allowed the production of a single integrated genetic map by merging mapping data from different crossing lines. In particular, this map includes 515 loci based on segregation data from several crosses simultaneously analyzed with a multipoint maximum likelihood method. This kind of integration allows one to have an overview of the distribution of SSR markers and to individuate as many transferable markers as possible within a single genetic map (Doligez et al., 2006). A separate study took advantage of three segregating populations obtained by crossing Syrah x Pinot noir, Syrah x Grenache and Cabernet Sauvignon x Riesling to compile an integrated genetic map including 1.134 markers (350 AFLP, 332 BESs, 169 ESTs, 283 SSRs), assessing also the transferability of SNP markers among the five parental cultivars (Vezzulli et al., 2008). A similar work was carried out by Vervalle et al. (2022) with the study of three mapping populations: Cabernet Sauvignon x Corvina Veronese, Riesling Weiss x Cabernet Sauvignon and Deckrot x G1-7220 (a table grape originated from Black Rose x Muscat Seedless). Based on those, a reliable reference integrated SNPs map was built up, representing the most saturated high-density integrated genetic map compiled so far for grapevine. The practical implications of a densely covered integrated map are related to the identification of markers spreading in any specific region of interest for positional gene cloning or MAB, but also to the detection and comparison between QTLs of important phenotypic traits. In this regard, Nicolas et al. (2016) performed an association study on a panel of 279 cultivars, giving an estimation insight of linkage disequilibrium and showing how a subset of polymorphisms can be useful to rationally choose phenotypic traits that can be evaluated in trials, knowing their heritability a priori. In this regard, an association test simulation is very useful to depict traits heritability, minor allelic frequency, locus differentiation and QTLs effect, obtaining an efficient association overview. Genetic maps enabled the study of QTLs closely related to phenotypic traits of interest, with important implications and applications which allow to speed up grapevine breeding process and to carry out varietal selection in a precise and targeted manner. Grapevine MAB largely relies on genetic maps and the development of denser genetic maps equips the breeding system with more effective tools, resulting in benefits that directly affect the efficiency of the entire process of varietal constitution.

### 4.2 Introgression of traits linked to abiotic stress resistance for varietal constitution

Here we shift our attention towards abiotic stresses and agronomic performances, while omitting the chapter dedicated to breeding for tolerance and resistance to biotic etiological agents, exhaustively reviewed in Pirrello et al. (2023). More than scions, above all rootstocks undergo a process of breeding and selection to attain this kind of desired traits. If from one side scion breeding is more focused on quality and productivity improvement, on the other hand rootstock genotypes may be enhanced in drought or mineral deficiency/toxicity tolerance. The need to implement a breeding system for rootstock varieties emerged in 1868, when Phylloxera was threatening to wipe away European viticulture. The solution was finally individuated in grafting the aerial part of European grapevine cultivars, featured by high oenological traits, on American varieties root system, resistant to the pest. Phylloxera resistant rootstocks immediately appeared inadequate to cope with European lime soils. In this regard, the first approach employed to address this challenge involved the discovery of *Vitis cinerea* Engelm var. *helleri* in Texas, a wild relative which showed high tolerance to calcareous soils. The problem of this species is that it is featured by a poor rooting ability. For this reason, several crosses were performed with other *Vitis* species in order to merge tolerance to calcareous soils and satisfactory root development (Töpfer et al., 2011). Thus, it was clear that the breeding had to cover a pivotal role for adapting American rootstocks to European field conditions. The Era of rootstocks began literally saving European viticulture. In general, rootstock breeding aims to improve a well-defined subset of performance traits, i.e. lime tolerance which on turn prevents iron chlorosis on calcareous soils, drought tolerance to ensure a correct berry development, the capacity to establish a satisfactory root system, and finally the affinity between rootstock and scion (Töpfer et al., 2011). A further impulse in this regard was achieved by Zsigmond Teleki, a Hungarian winegrower who analyzed about 40,000 plants of *V. cinerea* Engelm var. *helleri* grown in calcareous condition. The most promising genotypes were afterwards used as starting catchment basin to develop a real milestone of rootstock breeding, or rather Kober 5BB variety (Töpfer et al., 2011). A special mention should be given to M series rootstocks, developed in the past few decades by the University of Milano, in Italy. The aim was to select new grapevine rootstocks able to cope with climatic changes. In particular, the researched traits were a more efficient water uptake (pursued with both a reduced vigor to contain transpiration and drought resistance), enhanced tolerance to salt stress and iron chlorosis, and better soil nutrients absorption (Porro et al., 2012). In detail, the four genotypes developed were ‘M1’ [‘106/8’ (*V. riparia* × (*V. cordifolia* × *V. rupestris*)) × ‘Resseguier n°4’ (*V. berlandieri*)] selected for resistance to Fe chlorosis and soil salinity, and to reduce vigor; ‘M2’ [‘Teleki 8B’ (*V. berlandieri* × *V. riparia*) × ‘333 E.M.’ (*V. vinifera* × *V. berlandieri*)] for high K and Mg uptake efficiency and resistance to Fe chlorosis; ‘M3’ [‘R 27’ (*V. berlandieri* × *V. riparia*) × ‘Teleki 5C’ (*V. berlandieri* × *V. riparia*)] for reduced vigor; and ‘M4’ [‘41B’ (*V. vinifera* × *V. berlandieri*) × ‘Resseguier n°4’ (*V. berlandieri*)] for high resistance to water stress and soil salinity (Porro et al., 2012). It is pivotal to point out that the enhanced drought tolerance of M4 was elucidated at the molecular level. Through genetic and transcriptomic comparison with the commercial rootstock 101.14, M4 showed a greater activation of resveratrol and flavonoid biosynthetic pathways, respectively, in roots and leaves, under water stress conditions. The improved resistance to water stress is imputable to a greater accumulation of these compounds which are characterized by a high antioxidant capacity (Corso et al., 2015). In general, drought resistance is a research objective in breeding, and this very complex trait involves variables linked both to the roots and to the aerial part of the plant. Due to the grafting practice, the final performance is a combined outcome of scion and rootstock behaviors. The scion contribution involves the ratio between photosynthesis rate and transpiration rate, which in turn is a function of stomatal conductance and leaf water potential (Eibach and Töpfer, 2015). In this regard, a GWAS analysis performed on 100 *Vitis* spp. accession highlighted 24 significant marker-trait associations along various stages of drought-stress and 13 candidate genes with a feasible role in drought response (Trenti et al., 2021), pointing out a coordinated action between scions and rootstocks for water use regulation. Another main issue related to grapevine abiotic stress is the frost tolerance in winter, as well as in case of late frost occurrences (De Rosa et al., 2021). A significant resistance to winter minima was described for wild relatives originating from strong continental climate conditions, e.g. *V. riparia* and *V. amurensis*, but also in some varieties of *V. vinifera*, such as Riesling (Eibach and Töpfer, 2015). On the other hand, cold-hardy varieties are often paradoxically more vulnerable to spring frost occurrences due to early bud break dates (Ferguson et al., 2014). The evaluation of these traits during breeding programs is complicated by the impossibility of predicting and observing natural occurrences of winter and spring frosts within a specific environment, which can only be simulated by freezing buds artificially. In this regard, it is interesting to notice the application of artificial frost procedures together with OIV frost resistance descriptors, to some introgression lines of *V. vinifera* to study complex frost resistance traits from *V. rotundifolia* Michx., highlighting the importance of traditional breeding both aimed to the creation of new rootstock and productive cultivars, and to the study of grapevine genetics (Volynkin et al., 2021). A study performed on *Vitis amurensis*, which among *Vitis* species in the coldest tolerant, individuated 17 different genes involved in cold signal transduction, suggesting a different mechanism between plant response to chilling temperature and to freezing conditions (Wang et al., 2021a), providing in this way a valuable genetic resource for grapevine breeding.

## 5 Innovative approaches to accelerate varietal selection in grapevine

CBTs still play a significant role in grapevine breeding, even if their implementation demands laborious operations including manual emasculation, pollen collection and hand pollination. Until recent times, promising individuals were mainly selected based on their phenotype and performance in the field. However, due to the particularly long breeding cycle of grapevines, a time frame spanning decades needs to be taken into account for the selection of a new cultivar (Eibach and Töpfer, 2015). The employment of genetic markers for early screening of genotypes of interest (mentioned above as MAB) improved the efficiency of selection, although genotyping thousands of individuals has remained prohibitive for a while. As a major drawback, marker-based QTL analysis proved inefficient in disentangling complex traits influenced by many genes with small effects or predicting allele effects in new, different environments. As the manifestation of climate change increases the urgency to produce resilient cultivars, innovative approaches are required to overcome the main obstacles in grapevine’s breeding. The natural genetic variability within the genus *Vitis* has already proved itself useful to address the issue of resistance against biotic (Schwander et al., 2012; Sapkota et al., 2023) and abiotic stressors (Liu et al., 2016). This leads to hypothesize that the solution for several climate change-related threats may exist within this genetic reservoir. For this reason, the introgression of genes from other *Vitis* species to cultivated grapevines represents a compelling strategy to face future challenges. Furthermore, effective solutions to the caveats of genetic transformation in clonally propagated perennial trees are paving the way to the application of NBTs, such as genome editing. This technique enables the generation of minor insertions or deletions, basically resulting in gene knockouts, but it can also allow for allele substitution and precise insertions, offering the potential to accelerate cultivar improvement in several fruit crops (Najafi et al., 2023). The CRISPR/Cas9 is currently the more cost-effective and efficient system adopted for genome editing, and it has been proved adaptable for woody plants. Several reviews have recently summarized the major outcomes of NBTs use in fruit plants, including grapevines (Campa et al., 2023; Nerva et al., 2023). In the context of promoting a climate-smart viticulture, noteworthy successes have been achieved with grapevine plants edited for cold resistance (Wang et al., 2021b), or water stress tolerance due to the inactivation of *VvEPFL9-1* in a table grape variety via CRISPR/Cas9 (Clemens et al., 2022), and all efforts aimed at obtaining grapevine plants resistant to pathogens (Pirrello et al., 2023). As expected, most of them were obtained by targeting susceptibility genes in host grapevine plants; this approach is considered more straightforward and is generally thought to enhance what is known as recessive resistance (Schenke and Cai, 2020), which is expected to be more slowly overcome by pathogen evolution.

Precise and predictable knock-in of advantageous genes from other species (transgenesis) or from the gene pool of the same species (cisgenesis) is another, less mentioned, but still crucial application of the CRISPR/Cas9 system. These possibilities are eliciting a resurgence of interest in genetic engineering due the reduced risks associated with potential unintended modifications of the host genome and to groundbreaking methods that promise to surmount the widely recognized challenges of grapevine regeneration after genetic modification (Campos et al., 2021). Nonetheless, while these remarkable biotechnological advancements are noteworthy, it is important not to overlook the continued significance of quantitative genetics in providing predictive models for genotypic values in breeding. This holds true as empirical methods based on the combination of high throughput genotyping with simulation tools gain prominence, particularly in the context of crop species characterized by rapid generational turnover, where the concept of genomic selection (GS) has already come into play, anticipating what could represent a new frontier for the fruit tree community.

### 5.1 Speeding up grapevine’s *in vitro* regeneration

As mentioned above, the ability to regenerate plants from transformed tissues is still one of the main pitfalls also in the production of transgenic and edited grapevine plants.

Plant regeneration is achieved mainly via two methods: shoot organogenesis (SO) and somatic embryogenesis (SE); SO implies the formation of new plant organs in response to different ratios of hormones directly from an explant or indirectly from calli (Su and Zhang, 2014), SE entails the formation, directly from explants or indirectly from calli, of embryo-like structures from dedifferentiated totipotent embryonic stem cells. While SE requires lengthy periods to complete, SO is faster but also characterized by higher chances of chimerism after the transformation (Nuzzo et al., 2022). SE represents the preferred method for regeneration of woody plants, for conservation or mass propagation, as well as genetic improvement purposes (Guan et al., 2016) and is most exploited for grapevine regeneration with varying efficiency depending on species, cultivar and starting tissue. Anthers and ovaries represent the most suitable explants, obtainable at flowering or from shoots sprouted under controlled conditions (Nuzzo et al., 2022). A recent improved SE protocol tested on several cultivated varieties and rootstocks, using flower tissues as starting materials, detected the highest embryogenic efficiency in whole flowers of hybrid rootstocks 110 Richter (*V. berlandieri* Rességuier n. 2 × *V. rupestris* Martin) and 17.37 (*V. berlandieri* x *V. rupestris*). Somatic embryo regeneration was also obtained for recalcitrant cultivar Glera, although definitive answers on the best culture conditions for this variety are still missing (Capriotti et al., 2022). Further evidence was recently reported of enhanced regeneration rates using cotyledons derived from flower-induced somatic embryos as starting material in Thompson seedless and other cultivated varieties (Capriotti et al., 2023). SE was also successfully employed on immature seeds to rescue virus-infected cultivated varieties (San Pedro et al., 2017). Whole plants, including genome edited ones (Najafi et al., 2023), were also regenerated using embryonic-callus derived protoplasts as starting material (Bertini et al., 2019). Recently, a description of the molecular mechanisms regulating SE in grapevine was provided by comparing recalcitrant cv. Cabernet Sauvignon and competent cv. Sangiovese, highlighting that embryogenic competence is achieved at early stages of tissue culture and that DNA methylation profiles could be used as a marker of SE competence (Dal Santo et al., 2022). Successful regeneration via organogenesis using meristematic bulk (MB) tissues was also reported in grapevine, for both rootstocks and scions, including wine and table varieties (Xie et al., 2016; Sabbadini et al., 2019). Cultivar Thompson seedless showed the highest natural competence towards regeneration and transformation and was thus proposed as a model variety for functional studies (Sabbadini et al., 2019).

Notwithstanding these examples, *in vitro* regeneration remains a major hurdle in grapevine’s genetic improvement programs due to the recalcitrance of this species and the overall duration of the process, which requires several months. New innovative protocols to speed up the regeneration process have been recently tested on other plant species and could potentially be extended to grapevine in the future. One approach is based on the co-transformation of the highly conserved transcription factors GRFs (*Growth Regulating Factors*) and their co-factors GIFs (*GRF-Interacting Factors*), which form complexes playing a role in several developmental processes in *Arabidopsis thaliana*, including cell proliferation, organ growth and size, and reproductive competence (Kim et al., 2003; Lee et al., 2009; Lee et al., 2014). The expression of specific combinations of GRFs-GIFs into fusion proteins was found to increase the efficiency and speed of regeneration in wheat, triticale and rice (Debernardi et al., 2020). The expression of species-specific homologs improved somatic embryogenesis in maize, as well as organogenesis in sugar beet, sunflower and soybean (Kong et al., 2020). Lastly, higher regeneration efficiency was reported for both wild and cultivated lettuce (Bull et al., 2023). Nine GRFs were recently found within the grapevine genome, with one, namely *VvGRF7*, responsible for the increased growth when overexpressed in *A. thaliana* (Hu et al., 2023). No evidence on the effects of co-transformation of GRFs-GIFs is currently available in grapevine and its potential remains to be tested. Another promising technique was recently described by Nasti et al. (2021), namely Fast-TrACC (*Fast Treated Agrobacterium Co-Culture*), as a tool for testing the best combination of transformation reagents in a transient and rapid way in *Nicotiana benthamiana*. Fast-TrACC represents the evolution of a previously developed method, AGROBEST (*Agrobacterium-mediated enhanced seedling transformation*) (Wu et al., 2014), used for transient expression of transgenes in *A. thaliana*. Both methods make use of specific growth media to induce *vir* genes expression, thus improving T-DNA transfer. Fast-TrACC was successfully used for delivering transgenes in potato, tomato, pepper and eggplant (Nasti et al., 2021). Genome editing reagents and DRs (*Developmental Regulators*) were also delivered to seedlings using Fast-TrACC to produce transgenic and gene-edited shoots of *N. benthamiana in vitro*, ultimately resulting in the production of whole gene-edited and transgenic plants, able to transmit the modifications to their progeny (Maher et al., 2020). A second approach was described, and named direct delivery (DD), consisting in the application of DRs to soil-grown plants at the wound site following the removal of a meristem. The formation of transgenic, or gene-edited, shoots was reported in *N. benthamiana* (Cody et al., 2023). While the formation of transgenic shoots in a shorter time compared to traditional regeneration was reported in tomato, potato and grapevine (Maher et al., 2020), the regeneration of whole grapevine plants still remains elusive.

### 5.2 Overcoming the phenotyping burden and long generational turnovers: genomic selection and prediction

CBTs rely on phenotype observation to select individuals of interest within a program. As already mentioned, the overall process translates into a lengthy endeavor when applied to woody perennials. MAB is effective in speeding up the process when it comes to the selection of traits associated with one or few genes, but that is rarely the case with abiotic stress resistance. In this context, GS represents a desirable alternative which estimates the individual effects of all markers distributed in a genome, whose additive sum is used to calculate an individual’s genomic-estimated breeding value (GEBV) (Budhlakoti et al., 2022). This approach takes advantage of data collected on previously analyzed populations, making the selection of valuable genotypes swifter. As a matter of fact, GS implies a training step consisting in phenotyping and high-density genotyping of populations to produce and calibrate a statistical model. Subsequently, it is applied to a target population to predict its breeding potential, solely based on its genotypic characterization. This reduces breeding times and costs, due to genotyping becoming more and more cost-effective in recent times. However, the prediction power of a model is directly influenced by the composition of its training population, making the choice of the parental genotypes crucial. Moreover, the heritability of each trait and the interaction between genotype and environment (G×E) also influence the accuracy (Budhlakoti et al., 2022). Models can be family-specific, with individuals deriving from the same cross being used in both training and target populations. These are generally highly accurate in terms of within-family predictions (Jacobson et al., 2014). Alternatively, genotypes unrelated to the training individuals can be included in the target population. In this scenario, individuals of varying genetic background should be included in the training population to allow ancestral relatedness to be present and useful. The accuracy of such models is usually lower compared to family-specific models (Lorenz et al., 2015) and very low predictive ability is found in models applied to a completely unrelated target population (Lehermeier et al., 2014; Lorenz et al., 2015).

The use of GS analysis increases the role of statistics in quantitative genetics, introducing a new paradigm for the required expertise of a breeder. Indeed, linear regression models play a fundamental role in GS and genomic-enabled predictions. Some of the most commonly used models for GS include the best linear unbiased prediction (BLUP), the ridge regression (RR), the least absolute shrinkage and selection operator (LASSO), and the elastic net (ENET), including their many variants. Linear mixed models (LMM) are also an active area of research for fitting GS models and involve best linear unbiased estimation (BLUE) of fixed effects (such as fixed environmental conditions) and BLUP of random effects (random genetic effects). The mixed-model framework has demonstrated its effectiveness in analyzing the phenotypic and SNP data that are regularly produced within a breeding program (Caamal-Pat et al., 2021).

GS via genomic prediction (GP) has been employed in both animal and plant breeding (Daetwyler et al., 2013), including numerous crops of economic relevance such as *Solanaceae* (Tong et al., 2022), cereals, legumes (Krishnappa et al., 2021; Kaler et al., 2022) and other horticultural crops (Hernandez et al., 2020; Liu et al., 2021). GP was also applied to fruit crops (**Table 2**) including apple (Roth et al., 2020; Minamikawa et al., 2021), apricot (Nsibi et al., 2020); passion fruit (Viana et al., 2017), pear (Minamikawa et al., 2018), peach (Li et al., 2023), banana (Nyine et al., 2018), blueberry (de Bem Oliveira et al., 2020), strawberry (Yamamoto et al., 2021), cranberry (Covarrubias-Pazaran et al., 2018), oil palm (Cros et al., 2015) and *Citrus* spp. (Minamikawa et al., 2017).

**Table 2:** Application of genomic prediction to woody perennial fruit crops.

Species	Techniques	Traits	Reference
Apple	GS; GS + GWAS	Fruit texture; fruit quality	(Roth et al., 2020; Minamikawa et al., 2021)
Apricot	GS	Fruit quality	(Nsibi et al., 2020)
Banana	GS	Agronomic traits, disease resistance, fruit traits	(Nyine et al., 2018)
Blueberry	GS	Fruit quality	(de Bem Oliveira et al., 2020)
Citrus spp.	GS + GWAS	Fruit quality	(Minamikawa et al., 2017)
Cranberry	GS	Yield and fruit weight	(Covarrubias-Pazaran et al., 2018)
Oil palm	GS	Yield	(Cros et al., 2015).
Passion fruit	GS	Fruit quality and yield	(Viana et al., 2017)
Peach	GS; GS + GWAS	Yield, fruit quality; Agronomic traits, VOCs	(Biscarini et al., 2017; Li et al., 2023)
Pear	GS + GWAS; GS + GWAS	Fruit quality, disease resistance, growth traits	(Minamikawa et al., 2018; Kumar et al., 2019)
Strawberry	GS	Vegetative and fruit-related traits	(Yamamoto et al., 2021)

GS, genomic selection; GWAS, genome-wide association studies.

Few studies are available regarding GP in grapevine (**Table 3**). A first pioneer study compared the predictive ability of GS alone or combined with GWAS in simulated populations of grapevine (Fodor et al., 2014). The simulated scenario included four training populations, corresponding to the three main genetic subpopulations which emerged following domestication (Table-East, Wine-East and Wine-West), a core collection representing the entire genetic diversity with minimal redundancy, and four breeding populations. This work pointed out how predictive ability is reduced when the breeding population is genetically distant from the training one, and that the highest accuracy is reached combining GS and GWAS using a core collection as training population (Fodor et al., 2014). GS was investigated a few years later in an actual breeding context in a table grape bi-parental cross and provided greater efficiency than QTL analysis for the inference on the genetic contribution of several marker loci for the selection of agronomic traits of interest (Viana et al., 2016). GS was also performed in association with GWAS to evaluate the association between already available historical phenotypic data from 580 V*. vinifera* accessions within a germplasm collection, and their newly recovered genotypic markers. This study allowed the identification of several loci targeted during grapevine domestication and suggests an association between a new locus and berry characteristics (Migicovsky et al., 2017). More recently, GP was used to focus on traits related to climate change adaptation, such as drought-related responses (Brault et al., 2021) and, in association with GWAS, to explore indirect defense phenotypes against mites (Laplante et al., 2021). GP and QTL detection were performed on a large diversity panel comprising 279 genotypes phenotyped for several years, allowing for a greater understanding of the genetic architecture of several target features, including biochemical, phenological, morphological, agronomical and stress-related traits (Flutre et al., 2022). Lastly, Brault et al. (2022b) endeavored to evaluate the potential for GP in grapevine by focusing on the parameters affecting the predictive accuracy of a model across populations, which more closely resembles what would take place in a breeding program. Fifteen traits were phenotypically measured in a half-diallel with 10 bi-parental crosses, and a diversity panel not including the five parental genotypes used in the cross. GP was applied within three scenarios: within the half-diallel population, using 90% offspring as training population and 10% as target population; using the diversity panel as training population and each half-diallel as target; using the three half-sibling populations as training population for each half-diallel cross as target. Predictive ability was significant for some traits, thus introducing the possibility of extending this model to other densely genotyped individuals not included in the half-diallel or diversity panel, potentially also unphenotyped (Brault et al., 2022b).

**Table 3:** Application of genomic prediction to grapevine.

Techniques	Populations	Traits	Reference
GS + GWAS	Simulated population of 3000 individuals	Simulated traits	(Fodor et al., 2014)
GS	*Vitis rupestris* × *Vitis arizonica*/*girdiana*	Agronomic traits	(Viana et al., 2016)
GS + GWAS	580 *V. vinifera* accessions	Sex, skin color, Muscat aroma	(Migicovsky et al., 2017)
GS	Pseudo-F1 progeny of *V. vinifera* cvs. Syrah and Grenache	Drought-related and simulated traits	(Brault et al., 2021)
GS + GWAS	399 *V. vinifera* accessions	Mites defense	(Laplante et al., 2021)
GS	Half-diallel with 10 bi-parental crosses	Yield, berry, phenology and vigour traits	(Brault et al., 2022b)
GS + GWAS	Diversity panel of 279 V*. vinifera* accessions	Stress-related traits	(Flutre et al., 2022)

GS, genomic selection; GWAS, genome-wide association studies.

Finally, it is important to note that the increased use of high throughput genotyping and phenotyping approaches makes the development of new algorithms, capable of handling very complex datasets, a necessity for future applications. In this regard, machine learning (ML) approaches, which efficiently manage complex genotypic and phenotypic training data to reach high prediction power, stand out as a new direction for the future. Common ML algorithms, such as random forest or artificial neural networks, can be applied to manage biological problems involving big amounts of data, including increasing phenotype prediction of traits characterized by a complex genetic structure using intermediate phenotypes such as DNA and RNA sequences (Wang et al., 2020). ML models are considered more apt than classic linear GS models, such as BLUP, in detecting non-linear relationships within data, often the case for plant-environment interactions. On the other hand, the high plant phenotypic plasticity exhibited in different environments and conditions remains one of the main challenges for ML models performance (Danilevicz et al., 2022). As a matter of fact, up to now ML algorithms applied in GS did not exhibit a significant upper hand when compared to linear mixed models and the general consensus is that no algorithm can perform best in every context (Azodi et al., 2019). For example, random forest performed best for plant height prediction in rice, while being outperformed by linear models for other traits (Spindel et al., 2015) and performed worst in wheat for different quality traits (Battenfield et al., 2016).

However, the true potential of ML may be unravelled in time by improving the thoroughness of training data, which must include phenotypic data collected in a range as wide as possible of conditions and environments. Moreover, a major aspect in which ML models differ from, and possibly surpass, classic GS models, is their higher explainability. Explainability, defined as the ability to explain how a model works and makes predictions even after it has been trained (Santorsola and Lescai, 2023), can provide information on the genomic sequences that contribute to the observed phenotypic variations. This is relevant for breeding programs and for the overall understanding of biological processes (Danilevicz et al., 2022). Finally, epistasis, a form of interaction between genes, or genomic imprinting, coinciding with complete inactivation of maternal or paternal alleles, are sources of non-additive genetic variation better integrated in ML approaches than classic GS models (Varona et al., 2018). As the situation stands, ML represents a compelling perspective for application in GS, despite the need to improve training datasets completeness by, for example, sharing privately stored genotypic and phenotypic data (Spindel and McCouch, 2016).

Thus, GS approaches are surrounded by increasing interest also in the viticulture community and the delay of its application to this field compared to the animal breeding scenario is compensated by many more tools and more sophisticated models being available nowadays relative to the first introduction of the GS methods in early 2000s. Although the focus of this examination has been centered on resilient traits, it should be noted that the traits that could benefit from the shortcuts of GS also include those related to wine quality and other oenological features that can be evaluated much later in the life cycle of the plant.

### 5.3 Beyond genotyping: phenomic prediction

The concept of phenomic selection was first introduced in 2018 when Rincent et al. (2018) proposed to use so-called endophenotypes, i.e. molecular phenotypes composed by small RNAs, transcripts and metabolites (MacKay et al., 2009), captured using near-infrared spectroscopy (NIRS) to non-destructively predict the unknown phenotype of, virtually, any plant species. The use of NIR signals, represented by emission or reflection of light from the sample, leans on the presence of chemical bonds in the analyzed tissue, supposedly connected to its endophenotype, allowing a barcoding of the sample. For this reason, NIR spectra have been proposed as a genetic marker for the discrimination of species or even varieties (Posada et al., 2009; Lang et al., 2017). A training population is required similarly to GP, with choice of tissue, environment and probe type representing additional variables affecting the predictive ability (Robert et al., 2022). Nevertheless, the use of such an approach to support breeding programs represents a compelling possibility, since, while having decreased in recent years, the costs of genotyping can still be prohibitive when large numbers of individuals need to be screened. NIRS remains in fact a high-throughput, low-cost option compared to omic approaches (Rincent et al., 2018). Up to now, very few examples of the application of phenomic prediction as a proxy for complex traits in plant breeding are available, mostly in assistance of horticultural crops and cereals breeding using leaves or kernels (Lane et al., 2020; Zhu et al., 2021; Jackson et al., 2023). High predictive accuracy was achieved in soybean, with phenomic prediction appearing less bound to relatedness than GP (Zhu et al., 2021). Similar conclusions were reached in winter wheat using hyperspectral imaging to predict yield (Jackson et al., 2023). The possibility of using NIR data to evaluate independent populations also appeared possible in maize (Lane et al., 2020). Almost no evidence on the use of phenomic prediction is available for woody perennials. A work carried out in Eucalyptus suggests NIR spectra to be a promising tool for the study of the genetic control of wood-related traits (Hein and Chaix, 2014). On the other hand, NIR-based prediction varied in its efficiency depending on the considered trait in poplar (Rincent et al., 2018). Very recently, promising results were collected in grapevine comparing GP and phenomic prediction accuracy on a diversity panel and a half-diallel cross (Brault et al., 2022a). Fifteen traits related to berry composition, cluster morphology, phenology and vigor were evaluated collecting NIR spectra from wood and leaf tissues. Prediction accuracy appeared generally higher for GP in both contexts, although differences appeared very low or, for some traits, phenomic prediction even performed best. Regardless, a joint use of phenomic and genomic prediction performed best (Brault et al., 2022a).

Overall, phenomic prediction remains a fairly unexplored field that, while presenting itself as a promising path in research contexts, needs to be tested in more practical breeding endeavors to truly reveal its potential.

## 6 Concluding remarks

Climate change represents a multifaceted opponent to viticulture as it has been traditionally practiced. While research on grapevine stress resistance has made strides forward in recent years, providing more effective solutions to counteract biotic threats among others, major efforts are still necessary to elucidate the genetic basis of complex traits on which the impact of a changing climate is already apparent, such as drought responses or phenological dynamics. These traits are in fact influenced by multiple environmental factors, spanning from temperature changes to hydration conditions, to soil characteristics, many of which have exposed a deep complexity, raising the bar that quantitative genetics must endeavor to surpass. Despite significant advances enabled by the blooming of grapevine genomics and the exploitation of increasingly efficient molecular marker technology in marker assisted breeding, several years are still required to develop new competitive varieties improved in traits such as these. Two primary key strategies, in addition to well established practices, are currently being developed to quickly boost variety innovation in viticulture. One is rather conservative in terms of exploitation of genetic resources, albeit sophisticated for the technology involved, because it aims at protecting historical genotypes associated with consolidated wine tastes by introducing small and focused genetic changes in existing genomic backgrounds. This approach requires reinventing plant genetic transformation by exploiting the unprecedented tools now available for genetic engineering (e.g. genome editing) and tackling the problem of plant *in vitro* regeneration more resolutely and less empirically than ever before. Drawbacks to this approach include the need for significant and prior research efforts to identify genetic targets to aim at as well as the uncertainty lingering over legislative and public acceptance issues related to genetically modified organisms. The second strategy builds upon the methods of genomic prediction initially established at the beginning of the current millennium to speed up the selection process. This approach reduces the toll of multiple generation phenotyping and tackles the challenges of quantitative genetics more empirically through an empowered utilization of anonymous genetic markers for phenotype prediction. The caveat of such an approach when applied to perennial fruit trees like grapevine is the need for an initial effort, potentially very demanding, to genotype and phenotype a training population and develop a statistical model to predict the breeding value of incoming progenies based on multi-marker information.

New breeding technologies and genomic selection also address distinct goals that are defined by the different complexity of the genetic basis of the traits amenable to improvement. Polygenic traits are less treatable with NBTs, which instead are optimal in dealing with monogenic characters. However, the two approaches are complementary and their integration could be achieved in a unique breeding effort where NBTs can optimize varieties improved by genomic selection or equip pre-breeding materials with protection against biotic challenges. Thus, a forward-looking attitude is needed to put together the resources necessary to start these breeding programs and establish the long lasting plant materials to be used in the next decades.

## Author contributions

GM: Writing – review & editing, Investigation, Writing – original draft. VDR: Writing – original draft, Writing – review & editing, Investigation. MM: Investigation, Writing – original draft, Writing – review & editing. RF: Investigation, Writing – review & editing. AA: Conceptualization, Funding acquisition, Supervision, Writing – review & editing. GB: Conceptualization, Funding acquisition, Supervision, Writing – review & editing. EP: Conceptualization, Funding acquisition, Supervision, Writing – review & editing. AV: Conceptualization, Funding acquisition, Supervision, Writing – review & editing. EDP: Conceptualization, Funding acquisition, Supervision, Writing – review & editing.
